# Diabetic ketoacidosis masked by both Euglycemia and a primary metabolic alkalosis

**DOI:** 10.1093/omcr/omae071

**Published:** 2024-07-13

**Authors:** Matthew F Hillock, Cierra Jarmon, Anastasia E Metropulos, Rachael King, Stefan Tchernodrinski, Daniel R Principe

**Affiliations:** University of Illinois College of Medicine, Chicago, IL, United States; University of Illinois College of Medicine, Chicago, IL, United States; Northwestern University Feinberg School of Medicine, Chicago, IL, United States; University of Illinois College of Medicine, Chicago, IL, United States; University of Illinois College of Medicine, Chicago, IL, United States; University of Illinois College of Medicine, Chicago, IL, United States; Northwestern University Feinberg School of Medicine, Chicago, IL, United States

**Keywords:** diabetes, euglycemic ketoacidosis, acid-base disorders

## Abstract

Diabetic ketoacidosis (DKA) is an acute, life-threatening metabolic complication of diabetes classically associated with hyperglycemia, metabolic acidosis, and ketosis. Though relatively uncommon, patients can also develop DKA with relative euglycemia, further complicating diagnosis. Here, we describe the case of a patient who presented with intractable vomiting secondary to diabetic gastroparesis. He was euglycemic, non-acidemic, and serum bicarbonate was within normal limits. However, labs were significant for ketonuria, an elevated anion gap, and an elevated beta-hydroxybutyrate. Given the high concern for euglycemic DKA in the setting of a competing primary metabolic alkalosis, he was transferred to the intensive care unit for intravenous insulin infusion and fluid resuscitation with significant clinical improvement and normalization of laboratory results. This serves as an important reminder that DKA can be masked by euglycemia as well as additional metabolic derangements, and should be suspected in any diabetic patient with an anion gap and/or ketosis.

## Introduction

Diabetic ketoacidosis (DKA) is an acute, life-threatening metabolic complication of diabetes. DKA is classically associated with hyperglycemia, metabolic acidosis, and ketosis [1]. Though DKA is classically characterized by blood glucose levels greater than 250 mg/dl, patients can also develop euglycemic DKA, which is defined by a blood glucose of less than 250 mg/dl in the presence of severe metabolic acidosis and ketonemia [2]. Though uncommon, euglycemic DKA is associated with several risk factors, most notably the use of sodium-glucose cotransporter 2 (SGLT2) inhibitors [3]. Here, we describe the case of a patient with euglycemic DKA that was masked by a competing primary metabolic alkalosis resulting in normal serum pH and bicarbonate levels. Hence, this case serves as an important reminder that DKA should be included in the differential diagnosis of any diabetic patient with an elevated anion gap and/or ketosis, regardless of serum glucose, pH, or bicarbonate levels.

## Case presentation

A 37-year-old man presented to the emergency department (ED) with nausea and vomiting for three days. His medical history was significant for hypertension and poorly controlled type 2 diabetes complicated by peripheral neuropathy, eschar of the left great toe, and several hospitalizations for gastroparesis. The patient had been admitted 8 times within the last six months with nearly identical symptoms, most recently two months ago. At each of his previous admissions, his symptoms resolved with IV fluids and pain medications. Each time, he was discharged home with GI and endocrinology follow-up, but declined further workup. At the present admission, the patient was somnolent and unable to fully cooperate with interview. When lucid, in addition to endorsing severe abdominal pain, he stated that he had three days of emesis and had been unable to tolerate any PO intake including his oral medications. He also stated that he is generally adherent to his empagliflozin and nighttime insulin (30 U Glargine), but is inconsistent with his mealtime insulin (5 U Aspart).

The patient was mildly tachycardic to 125 and hypertensive to the 160s/90s. Mucus membranes were dry and there was diffuse abdominal tenderness without guarding or rebound. Responses were delayed and the patient was only able to speak in short sentences. CT of the abdomen and pelvis was negative for cholecystitis or other acute process, and EKG showed sinus tachycardia. Urinalysis was significant for glucose > 1000 mg/dl, ketones > 100 mg/dl and complete blood count for a leukocytosis of 12.7 K/mm^3^. His basic metabolic panel noted hyponatremia of 128 mEq/l, hypochloridemia of 91 mEq/l, an anion gap of 23, and an initial blood glucose of 186 mg/dl. Venous blood gas was obtained demonstrating a pH of 7.37 and bicarbonate of 26 mmol/l, both within normal limits. Despite these values and relatively normal blood glucose, given the high clinical concern for DKA, we obtained a beta-hydroxybutyrate level which was elevated to 7.1 mmol/l.

Based on these results (summarized in Table 1), our team concluded that the most likely etiology of his symptoms was euglycemic DKA combined with a primary metabolic alkalosis due to severe recurrent vomiting. Though the possibility of a starvation ketosis could not be completely excluded, given the poor outcomes of untreated DKA, the patient was transferred to the medical intensive care unit for intravenous (IV) insulin infusion, constant electrolyte monitoring, and IV fluid resuscitation. He was also started on a lidocaine drip for pain management, which improved both his hypertension and tachycardia. The following morning, the patient’s anion gap had closed and beta-hydroxybutyrate returned to normal limits. The patient was now able to tolerate a clear liquid diet, his insulin drip was discontinued, and he was transitioned back to his basal insulin. His pain was well managed by a combination of ketorolac and acetaminophen, and the lidocaine drip was discontinued. He was transferred to the general medicine service, where he continued to show symptomatic improvement, and was discharged home in stable condition.

**Table 1:** Pertinent laboratory values

Component	Value	Reference Range
Blood Glucose	283 (H)	65–110 mg/dl
Blood Urea Nitrogen	36 (H)	6–20 mg/dl
Sodium	128 (L)	135–145 mmol/l
Potassium	4.1	3.5–5.2 mmol/l
Chloride	91 (L)	98–108 mmol/l
CO_2_ content	24	24–32 mmol/l
Calcium	9.6	8.6–10.6 mg/dl
Creatinine	1.39	0.50–1.50 mg/dl
Glucose	266 (H)	65–110 mg/dl
Anion gap	23 (H)	3–11 mmol/l
Estimated GFR	67	≥ 60 ml/min/1.73 m*2
Lactic acid	2.0	0.5–2.0 mmol/l
Magnesium	2.4	1.8–2.4 mg/dl
Color, urine	Light Yellow	Colorless, Light Yellow, Yellow
Clarity, urine	Clear	-
Specific gravity, urine	1.026	1.003–1.035
pH, urine	5.5	≤ 8.0
Protein, urine	20 mg/dl	Negative
Glucose, urine	> 1000 mg/dl (A)	Normal
Ketones, urine	> 100 mg/dl (A)	Negative
Bilirubin, urine	Negative	Negative
Nitrite	Negative	Negative
Urobilinogen, urine	2 mg/dl (A)	Normal
Blood, urine	Negative	Negative
Leukocyte esterase	Negative	Negative
Beta-hydroxybutyric acid	7.1 (H)	< 0.4 mmol/l
Glycosylated HgBA1c	8.5 (H)	< 5.7%
pH, mixed venous	7.37	7.33–7.42
PCO_2_, mixed venous	47	40–52 mmHg
PO_2_, mixed venous	61 (H)	25–40 mmHg
Base excess, mixed venous	1	−2–2 mmol/l
Serum Bicarbonate, mixed venous	26	22–26 mmol/l
Measured O_2_ saturation, mixed venous	87 (H)	60%–85%

Abbreviations: High (H); Low (L); Abnormal (A).

## Discussion

Euglycemic DKA is becoming increasingly prevalent, in large part due to the advent of SGLT2 inhibitors [4]. Though most patients with euglycemic DKA will recover with adequate treatment, this is dependent on prompt recognition as delayed diagnosis can lead to several complications including respiratory failure, hypovolemic shock, coma, or death [4]. As with hyperglycemic DKA, the diagnosis of euglycemic DKA is often challenging [5]. Though acidemia is a classic finding in DKA, patients can present with a normal or even alkalotic pH if there is a co-occurring metabolic or respiratory alkalosis [6]. Here, we describe a patient with euglycemic DKA in the setting of a competing metabolic alkalosis, resulting in a normal pH and serum bicarbonate. Though this has not yet been described in the literature, there is a similar case of a patient with euglycemic DKA in the setting of alkalosis, which delayed his diagnosis. Though this patient also had intractable vomiting and a competing metabolic alkalosis, unlike our case, this patient’s initial VBG was significant for a low partial pressure of carbon dioxide and low bicarbonate, strongly suggesting the presence of a concurrent respiratory alkalosis [7]. Hence, while most patients with euglycemic DKA will have acidosis, a normal or basic serum pH should not exclude the possibility of euglycemic DKA. Hence, any diabetic patient with ketosis and an anion gap may benefit from obtaining a beta-hydroxybutyrate level regardless of other laboratory values.

Importantly, there have been several instances in which the diagnosis of classical DKA has been obscured by mixed acid-base disorders or other metabolic derangements. Though diuretic use can confound serum pH in DKA, the most commonly reported cause for a normal or alkalotic pH in DKA is recurrent vomiting [6]. For example, a recent report describes the case of a 32-year-old type 1 diabetic who presented to the ED with intractable vomiting and fatigue. Workup was significant for hyperglycemia, metabolic alkalosis, and an anion gap. Given these findings, the patient was diagnosed with presumed “diabetic keto-alkalosis” due to DKA combined with a vomiting-induced metabolic alkalosis. As in our case, this patient improved following volume resuscitation and intravenous insulin [8].

Several other factors can also complicate the diagnosis of DKA, most notably cannabis use. A recent study of type 1 diabetics admitted for DKA found that cannabis users had significantly higher serum pH compared to non-users, which the authors attributed to cannabis hyperemesis syndrome [9]. However, a recent case report also describes a patient with hyperglycemic DKA with normal serum pH and bicarbonate in the setting of cannabis use without vomiting [10]. Hence, this warrants further exploration as a potential confounding factor in DKA. In summary, this case serves as an important reminder that, like classical DKA, euglycemic DKA can be masked by additional metabolic derangements and easily missed. Hence, DKA should be suspected in any diabetic patient with ketonemia and an anion gap, independent of serum pH or bicarbonate levels.

## Role of the funder

The funding agencies had no role in the design of the study; the collection, analysis, or interpretation of the data; the writing of the manuscript; or the decision to submit the manuscript for publication.

## Disclosures

The authors have no potential conflicts to declare.
